# Modeling Anomalous Moisture Transport in Cement-Based Materials with Kinetic Permeability

**DOI:** 10.3390/ijms21030837

**Published:** 2020-01-28

**Authors:** Zhidong Zhang, Ueli Angst

**Affiliations:** The Durability of Engineering Materials Group, Institute for Building Materials (IfB), ETH Zurich, 8093 Zurich, Switzerland; ueli.angst@ifb.baug.ethz.ch

**Keywords:** moisture transport, anomalous, cementitious materials, time-dependent permeability, drying and wetting, sorption isotherm

## Abstract

The durability of reinforced concrete structures is closely related to moisture state in cement-based materials. Therefore, it is crucial to develop moisture models that can accurately predict moisture state in the materials. However, many studies reported anomalous moisture transport in cement-based materials that cannot be well simulated by the conventional models. Several reasons have been investigated in the literature, such as the complex pore structure, chemical reactions with water, dimensional changes of the tested specimen, etc. Nevertheless, only a few models are able to capture the anomaly of moisture transport. This study viewed the main moisture transport coefficient—permeability—as a kinetic variable that depends on both the degree of moisture saturation and the contact time. The time-dependence was formulated by the decay (for drying) or growth (for wetting) functions. The saturation-dependence was calculated by the van Genuchten–Mualem (VGM) model. These functions were then implemented into a moisture transport model that was developed in previous studies. The proposed model was validated by experimental data and showed a good agreement for cement pastes that were dried or wetted in the hygroscopic range. Numerical simulation results were also compared with the simplified solutions to a fractional derivative model (FDM) of anomalous diffusion and the empirical Weibull function. We found that the solutions to the FDM cannot provide appropriate results. Weibull function performs as well as the proposed model, but the empirical function lacks physical meanings.

## 1. Introduction

Concrete is the most widely used man-made material in the world. In 2018, the global cement production was about 4.1 billion tons [1], equivalent to more than a half ton per person. Concrete is a mixture of cement, water, aggregates, and sometimes additives used to adjust the properties. When cement is mixed with water, the chemical reaction (so-called hydration) starts immediately, and eventually, the mixture becomes a dense solid, which contains a large number of pores whose sizes range from nm to mm. The presence of these pores can lead to the deterioration of concrete and corrosion of reinforcing steel bars (rebars). In most natural conditions, pores are partially filled with water depending on the surrounding environment. During the freeze/thaw process in the winter time, all pores in concrete are eventually filled with water. Damage to concrete, such as cracking and spilling, can occur in the sequential freeze/thaw process because of the volume expansion of freezing water. The most serious damage to the reinforced concrete structures is corrosion of rebars that leads to the function loss of reinforced concrete [2], such as structure collapse. In general, rebars are well protected by the passive layer due to the alkaline environment in concrete. However, CO_2_ in the atmosphere can diffuse into concrete, and reduce pH of pore solution; thus, the rebar would lose its protective environment. Partially saturated concrete favors carbonation as there are enough empty pores for CO_2_ diffusion and sufficient water for the reaction with the alkali ions. Another type of commonly mentioned corrosion is the chloride-induced corrosion. When the chloride content at the rebar surface exceeds a certain level (known as the critical chloride content) [3], the passive layer on the steel surface is destroyed, and pitting corrosion can happen. Chloride penetrates concrete with liquid water [3,4,5,6]. The process is accelerated in the drying-wetting condition [7].

Clearly, all above-mentioned degradation mechanisms of reinforced concrete structures are dependent on moisture in concrete [8]. Therefore, the appropriate moisture transport models are essential for predicting structures’ durability. Conventionally, a moisture transport model based on Fick’s law of diffusion, Darcy’s law or Richards’ equation is used to predict the moisture state in cement-based materials [9,10,11,12,13]. While these models can capture the main features of moisture transport for some materials, the derivations from these models for the cement-based materials are often reported in the literature. For instance, the anomalous moisture transport is found [14,15,16,17], which cannot be simulated by the conventional models [14,15,18,19].

In the early literature, the anomalous moisture transport was often reported for water capillary imbibition [14,15,20], in which one side of the specimen contacts with liquid water so liquid water is sucked into the porous material due to the capillary forces. If the measured mass change curve (Δm vs. square root of time $t^{1/2}$) does not obey a linear relation, it is viewed as anomalous capillary imbibition. When the moisture transport occurs in the hygroscopic range, the case that the measured mass change curves do not follow the curves calculated by Fick’s law has been also termed as anomalous moisture transport [16,17]. It needs to be emphasized that the diffusion coefficient in the commonly mentioned Fick’s law is generally treated as a constant. Anomalous moisture transport in cement-based materials can result from various reasons. Cement-based materials have a broad pore size distribution, ranging from micropores to macropores (0.5 nm to several hundred µm), which are classified as interlayer pores, gel pores, and large and small capillary pores [21]. Nevertheless, most of the moisture transport models are developed based on the single-porosity concept. To interpret anomalous moisture transport in cement-based materials, the concept of dual-porosity was introduced [19]. The calculated curves successfully captured the shapes of various anomalous mass loss curves. This concept was further extended to a dual-permeability model, which improves simulation results compared with the single permeability model [18]. Chemical interactions of water with hydration products could also be one reason. For liquid water imbibition, Hall et al. [14] argued that the anomalous water absorption in cement-based materials is caused by re-hydration of unreacted cement and dehydrated components of hardened cement pastes. Zeng and Xu [22] assumed reaction kinetics occurring along with moisture transport in cement pastes and employed a fractional kinetic model to represent anomalous drying. The third most mentioned reason is the change of microstructure of hydration products (mainly C-S-H (In cement chemistry: C-S-H = calcium silicate hydrate; CH = calcium hydroxide.)) during moisture transport. The macroscopically observed drying shrinkage is induced by the microscopic damages of pores due to the relatively large capillary force established during water evaporation [23]. The swelling associated with the water uptake was observed by either the environmental scanning electron microscope (ESEM) [14] or nuclear magnetic resonance (NMR) [24]. Both shrinkage and swelling result in the changes of pore structures.

Since the conventional models cannot describe the anomalous moisture transport, models developed on the basis of more sound theoretical backgrounds are needed. Two approaches are generally used to develop a mathematical model, phenomenological and mechanistic. The former one mostly employs the empirical equations to fit the experimental data. For instance, the above-mentioned two models (the dual-porosity model [19] and the fractional kinetic model [22]), which provide a certain physical meaning for the fitted parameters but are not developed with basis on the transport mechanisms. The mechanistic approach starts with the fundamental physical analysis of the studied phenomena, such as the Richards’ equation for moisture transport in unsaturated porous materials. The dual-permeability model is one example which was developed based on the different transport mechanisms in large and small pores [18]. To work for the anomalous capillary imbibition, models that are built based on Richards’ equation must be combined with empirical functions for the transport coefficients (diffusivity or permeability).

The fractional or fractal derivative (FDM) of diffusion models were reported to be able to well simulate anomalous water transport in soils [25,26]. However, in these models, the diffusivity is assumed to be constant (independent from water content), which is certainly not the case for cement-based materials. A recent study [27] employed an analytical solution of a simplified fractional derivative diffusion model, which was originally developed for the drying of foods [28] to fit the measured drying kinetics of concretes exposed to different drying conditions. In fact, this analytical solution is a variant of Weibull function with a scale factor (will be discussed later). This means that the development of such a model tried to start with the transport mechanisms but finally ended up with the phenomenological approach. Because of the difficulty to solve the fractional or fractal derivative models, in particular for the case that the diffusivity is not constant, an alternative method is to introduce a time-dependent variable [29,30,31]. In studies [29,30], the diffusivity was divided into two independent variables: one is a function of water content alone, and another is only dependent on time. The authors finally chose the power function for the time-dependent variable [29] and the exponential function for the water content-dependent variable [30]. The problems with the power function are that the diffusivity is infinite at time zero and that it will continuously drop with time without reaching a limited value. In a very recent study by Hall [31], an exponential time-dependent function was introduced for permeability to describe sorptivity measured during water capillary imbibition. The concept of time-dependent transport coefficient in these studies worked very well for capillary imbibition.

In the present study, we adopt the same concept of time-dependent transport coefficient for modelling moisture transport in the hygroscopic range. We view the permeability as a kinetic variable, which depends on both moisture content and contact time (see Section 4.1). These functions are then implemented into a Richards’ equation-based moisture transport model. The proposed model is then validated by experimental data and compared with the analytical solutions to a fractional derivative model of anomalous diffusion and Weibull function.

## 2. Results

### 2.1. Fitted Sorption Isotherm

**Figure 1:**
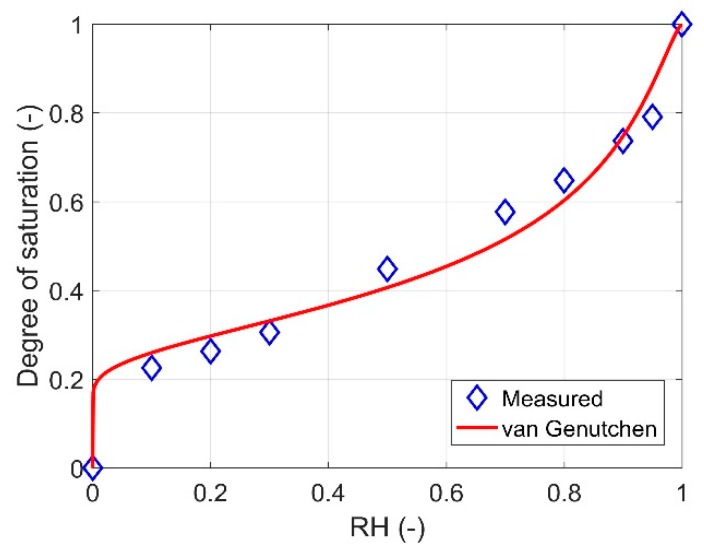
The desorption isotherm for the studied material. The isotherm was measured by DVS (data taken from [36]) and fitted by van Genutchen equation (Equation (9)).

The measured and fitted sorption isotherms by van Genutchen equation (Equation (9) in Section 4.1.1) are shown in Figure 1. When RH decreases from 1, the measured curve shows a sharp drop induced by emptying the large- and over-capillary pores. The second large drop is found between 50% and 30% RH. One argument is that this drop may be caused by measured water in capillary pores changing to in C–S–H pores [32]. Some studies believe that this is caused by cavitation of dissolved gas in pore solution [33,34]. However, the very steep sorption isotherm can also be caused by pore-blocking/percolation in a narrow range of pore necks [35]. There is no solid evidence to prove or disprove one of the hypotheses. These two drops result in the measured curve not being well fitted by van Genutchen equation.

### 2.2. Fitted Mass Change Curves

The mass change with time m(t) for the proposed moisture transport model (in 1D) was determined by the mass difference between the initial moisture saturation and the calculated moisture saturation at t.

(1) $$m\left(t\right)=\rho_{l}\phi A\overset{N}{\underset{i=1}{\sum }}\left|S_{i}\left(t\right)-S_{0}\right|\Delta x_{i}$$

where A [m^2^] is area of the specimen cross section; ϕ is the porosity; $\rho_{l}$ [kg/m^3^] is the density of liquid water; N is the total number of elements; i stands for a specified location (element number in numerical calculation), and $\Delta x_{i}$ is the size of element at the location i. $S_{i}\left(t\right)$ and $S_{0}$ are the saturation at time t and the initial saturation, respectively.

The mass change curves for two simplified solutions to the fractional derivative model (FDM) (Equations (23) and (25)) and the Weibull function (Equation (24)) are calculated based on the calculated MR (Equation (20) by the following equation.

(2) $$m\left(t\right)=m_{e}+\mathrm{MR}\;\times \;\left(m_{0}-m_{e}\right)$$

where $m_{0}$, m(t) and $m_{e}$ are the specimen masses at the initial state, during moisture transport and the final state, respectively. It is clear that $m_{e}$ must be determined by fitting the measured mass change curves.

**Figure 2:**
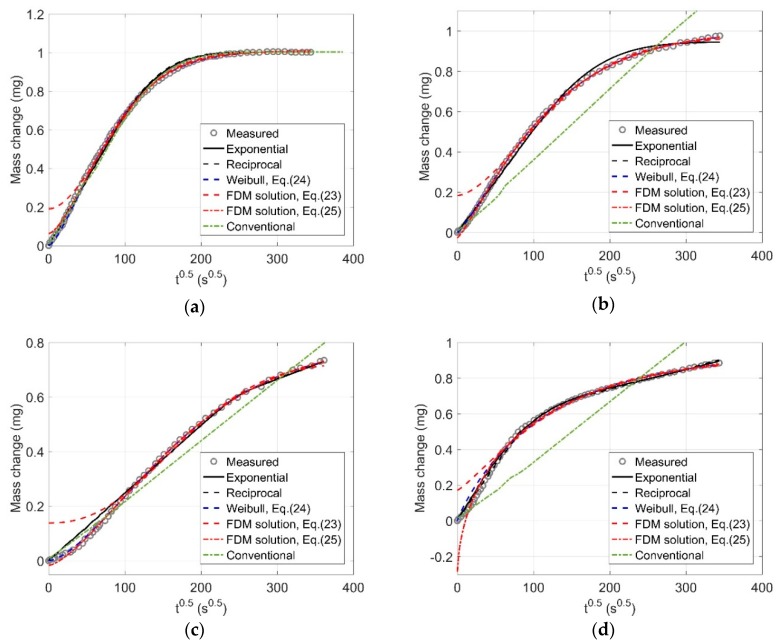
Measured and fitted anomalous moisture mass change curves for different drying or wetting conditions: (**a**) wetting at 50% RH from initial 60% RH; (**b**) wetting at 60% RH from initial 70% RH; (**c**) drying at 80% RH from initial 70% RH, and (**d**) drying at 80% RH from initial 90% RH.

The simulated mass change curves are compared with the measured ones in Figure 2. For the conventional model, permeability $K_{l}$ is the only parameter to be determined. All curves in Figure 2 are shown with $t^{0.5}$ as the x axis so that the difference amongst compared models can be better seen since the difference is mainly observed at the early stage of mass change.

The conventional model cannot capture the shapes of the mass change curves, except for Figure 2a. A mass change curve shows three stages: initial stage, transition stage, and later stage. At the initial stage, the linear curve of mass change vs. $t^{0.5}$ can be seen. At the later stage, the curve reaches a plateau if the porous material is rigid; however, if the microstructure of the material is altered, the curve may continuously increase with a low rate. Figure 2a shows a case without significant microstructure alteration; thus, the conventional model is able to provide good result. However, at the later stage, the curves for the other cases continue to increase with a lower rate than the initial stage, so the conventional model fails to simulate these curves.

The other models can well fit the late part of the curve. But for the initial part (roughly <100 s^0.5^), they show very big differences. All curves fitted by Equation (23) do not pass through the origin, which is due to the scale factor of $8/\pi^{2}$. If let t = 0, Equation (23) yields MR = $8/\pi^{2}$, which is above the origin. In a recent study [27], the scale factor was set as free parameter that is determined by fitting the experimental data (see Equation (25)). It indeed improves the fitting results, but there is still no rule to force the curve to pass through the origin. The starting point of the fitted curves by Equation (25) is either above or below the origin as shown in Figure 2. This violates the physical theory of moisture transport. Therefore, we conclude that both simplified solutions to the FDM are not able to provide reasonable results. If the scale factor is intentionally allowed to be 1, that is, the Weibull function, it can ensure the fitted curve passes through the origin and thus largely improves the fitting results.

The proposed model, either using the exponential function or the reciprocal function to calculate the time-dependent permeability $K_{t}$, is able to provide much better results than the two simplified solutions to the FDM, even better than the dual-permeability model proposed in our previous study [18]. Figure 2 as well as parameters in Table 1 shows that two functions for the calculation of $K_{t}$ provide almost the same results, suggesting that any decay or growth functions following the similar tendency could be used to calculate $K_{t}$.

In Figure 2, the proposed model and Weibull function provide very similar results as both of them can fit the experimental data well. However, Weibull function is a phenomenological model, and parameters in this model are not associated with any physical meaning. The proposed model was developed based on the mechanistic approach, but the drawback is that it requires more input data and a numerical algorithm to solve nonlinear equations. Therefore, researchers and engineers can choose the model depending on their aims. As concluded in the previous studies [18,19], if one wants to estimate the final mass of a specimen after drying or wetting (most likely needed in sorption isotherm measurements), the phenomenological models, such a Weibull or dual-Weibull equations [19], are the better choice because they are easy to apply and suitable for specimens with irregular geometries.

In Table 1, the calculated $K_{t}$ for wetting is higher than that for drying. This agrees with Mualem’s theory [37] that moisture movement during wetting can be faster than that during drying. The difference might also result from the fact that these measurements were performed on different specimens, as the previous study [38] has concluded that even for the same cement and mixture design, the measured permeability values on different cement paste/concrete specimens scatter in a large range, about one order of magnitude difference. We should also bear in mind that $K_{t}$ only represents the time-dependent permeability rather than the overall permeability. To calculate the overall permeability, $K_{t}$ needs to be multiplied by the saturation-dependent permeability $K_{s}$, which decreases sharply with the decrease of RH.

The kinetic rate constants of permeability ($K_{2}$) in Equations (15) and (16) indicate how fast permeability changes with time. It shows that $K_{2}$ values of wetting from 50% RH for the exponential function and drying from 90% RH for the reciprocal function are slightly higher than the other conditions. The kinetic rate constant of MR ($k_{1}$) in Equation (26) also shows that drying from 90% RH has the highest kinetic rate constant. However, the fitting accuracy for this condition is low as the curve starts at a point far below the origin. The high kinetic rate constant leads to the steep rise of the curve, but it cannot reflect the real kinetic rate constant of the measured curve.

## 3. Discussion

### 3.1. Apparent Diffusivity

**Figure 3:**
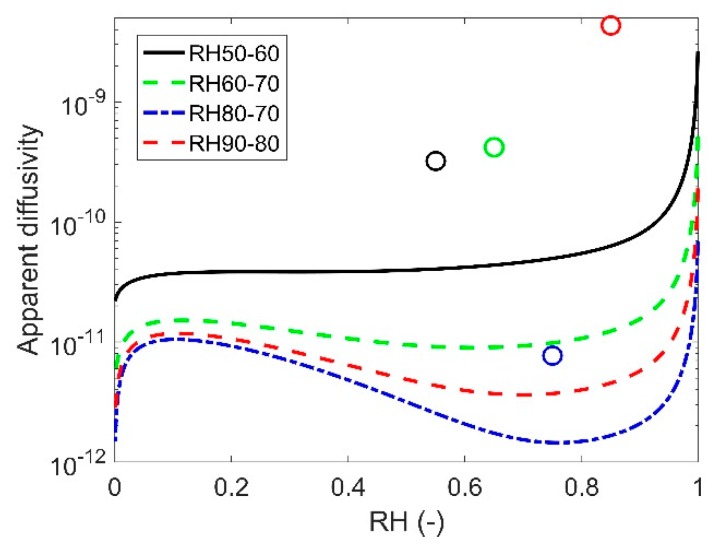
The apparent diffusivity calculated by the proposed model (lines) and Equation (3) (circles). In the proposed model, the contact time $t_{c}$ is taken as 1000s and $K_{t}$ is calculated by the exponential function. Each color corresponds to one condition.

The empirical solution to the FDM is involved by the apparent diffusivity. Since the size of the specimen is known, the apparent diffusivity $D_{f}$ can be calculated by rewriting Equation (22).

(3) $$D_{f}=k^{-\beta }{\left(\frac{\pi }{2L}\right)}^{-2}$$

where L [m] is the thickness of the planar specimen. The fitting parameters k and β for different drying and wetting conditions are shown in Table 1. They are used to calculate $D_{f}$, which is provided in the table as well. They are compared with the apparent diffusivity curves from the proposed model in Figure 3. For each condition, since the fitted parameters are different, the apparent diffusivity curve for each case can be drawn. 

Figure 3 shows that the apparent diffusivity curves decrease sharply from the saturated condition. The previous study suggested that this decrease is caused by the loss of liquid water [38]. With the further decrease of saturation, liquid water loses its continuity and then gradually the transport of water vapor becomes the dominant transport mode. The highlighted values of $D_{f}$ determined by Equation (3) are much higher than those from the proposed model regardless the drying or wetting condition. They may suggest that the FDM is not an appropriate model for the anomalous moisture transport in the studied material. But since Equation (21) is a highly simplified solution to the FDM, it may also suggest that Equation (21) cannot represent the actual solution to the FDM.

### 3.2. Validation Data

It has been argued that the measured mass loss alone is not sufficient to identify parameters in the moisture transport model, in particular for models with more than one parameter [18,39]. This is supported by a recent study [40], which has five parameters to be determined. Therefore, it needs at least the second set of experimental data to validate the moisture transport model, such as the monitored relative humidity evolution at given depths [40] or measured water content profiles at different exposure durations [18]. The remaining question is whether two sets of data are enough for a moisture transport model with more than two unknowns. For a two-phase model (liquid and vapor), it would be reasonable to have one parameter for each phase. If there is more than one parameter for each phase, it may cause less robust fitted parameters. Specimens for the experimental data used in this study were too small to measure water content profiles or to monitor RH evolution. The future study will be focused on the larger specimens to obtain enough data to further validate the proposed model.

## 4. Materials and Methods

### 4.1. Proposed Moisture Transport with Kinetic Permeability

#### 4.1.1. Conventional Moisture Transport Model

One of the earliest moisture transport models for unsaturated porous media is Richards’ equation published in 1931 [41]. Richards found that Darcy’s law, which was initially developed for saturated flow in porous media, also worked for water movement in unsaturated non-swelling soils. From the 1950s, the development of theoretical basis models according to the multiphase transport boomed [12,42]. The principle of multiphase, including liquid water, water vapor and dry air, was also applied to studies on mechanics and physics of porous media [10]. For moisture transport in cement-based materials, the multiphase model can be simplified as a two-phase transport model, just including liquid water and water vapor [11,43]. Assuming the quasi-equilibrium between liquid and vapor during the movement of moisture, the movement of two phases can be combined into one mass balance equation (in 1D form).

(4) $$\frac{\partial S}{\partial t}=\frac{\partial }{\partial x}\left(D_{v}\frac{1}{P_{v0}\phi }\frac{\partial P_{v}}{\partial x}\right)+\frac{\partial }{\partial x}\left(K_{l}\frac{1}{\eta_{l}\phi }\frac{\partial P_{l}}{\partial x}\right)$$

where S [−] is the degree of water saturation (the volumetric ratio of water and total pore space), $D_{v}$ [m^2^/s] is the diffusion coefficient of water vapor in the porous material; $P_{v0}$ [Pa] is the saturated vapor pressure; $P_{v}$ [Pa] is the vapor pressure; $K_{l}$ [m^2^] is the permeability of liquid; $\eta_{l}$ [Pa·s] is the dynamic viscosity of liquid water, and $P_{l}$ [Pa] is the liquid pressure.

The first term on the right-hand-side of Equation (4) represents the diffusion of water vapor which only occurs in the empty space of the pore network that is not occupied by liquid water. Note that the vapor diffusion in this study is different to the moisture diffusion in some literature [9,44], which did not distinguish liquid and vapor. Instead, they viewed moisture transport as a pure diffusion process. The second term on the right-hand-side of Equation (4) is the extended Darcy’s law where liquid water moves under the gradient of liquid pressure. Since two phases are always at equilibrium, saturation S and relative humidity $\mathrm{RH}\;\left(=P_{v}/P_{v0}\right)$ in the unsaturated porous media are interchangeable by using the water vapor sorption isotherm. Therefore, Equation (4) can be written as a transport equation similar to the standard Richards’ equation [43,44].

(5) $$\frac{\partial S}{\partial t}=\frac{\partial }{\partial x}\left(D_{a}\frac{\partial S}{\partial x}\right)$$

where $D_{a}$ [m^2^/s] is the apparent diffusion coefficient with both contributions of liquid water and water vapor. They are separable, and then, we have [38]

(6) $$D_{a}=D_{va}+D_{la}$$

with

(7) $$D_{va}=-{\left(\frac{M_{w}}{\rho_{l}RT}\right)}^{2}D_{v}\frac{P_{v0}\mathrm{RH}}{\phi }\frac{dP_{c}}{dS}$$

(8) $$D_{la}=-K_{l}\frac{1}{\eta_{l}\phi }\frac{dP_{c}}{dS}$$

where $D_{va}$ and $D_{la}$ are contributions of water vapor and liquid water to $D_{a}$, respectively. $M_{w}$ [g/mol] is the molar mass of water; R [J/K/mol] is the gas constant; T [K] is the temperature, and $P_{c}$ [Pa] is the capillary pressure. For a given RH, $P_{c}$ is determined by Kelvin’s equation.

To obtain sorption isotherm, the capillary pressure curve (also known as water retention curve for soils) must be known. Amongst various equations in the literature, the one proposed by van Genutchen [45] is often used (e.g., [11,38,46]).

(9) $$S={\left[\frac{1}{1+{\left(P_{c}/\alpha \right)}^{1/\left(1-m\right)}}\right]}^{m}$$

where α [Pa] and m are two parameters, which are generally determined by fitting the measured water vapor sorption isotherm.

The transport coefficients $D_{v}$ and $K_{l}$ are the two main variables that control the moisture transport. In general, vapor diffusion contributes to the mass transport much less than liquid water. However, if the material is exposed to low RH environment, the mass contribution of vapor significantly increases [18,37] because liquid water loses its continuity in the pore network, and it must evaporate and then can move to the environment in the form of vapor. Vapor diffusion depends on several factors, such as the available empty space and the structure of the pore network. The exponential equation or the reciprocal functions with S or RH for transport coefficients in the literature are often found to consider the effect of water content, but they generally ignore the effect of pore structure. By extending an expression proposed by Millington [47], we have one equation that can take into account all these factors [36].

(10) $$D_{v}\left(S\right)=D_{v0}\cdot \phi \left(1-S\right)\cdot \phi^{x_{D}-1}{\left(1-S\right)}^{x_{D}+1}$$

where $D_{v0}$ [m^2^/s] is the free vapor diffusion coefficient in air and $x_{D}$ [−] is a material parameter. For soils, the value of $x_{D}$ is about 4/3 [47]. However, for cement-based materials, this value largely overestimates vapor diffusion because of the high tortuosity. By calibrated with O_2_ and CO_2_ transport data, Thiéry et al., suggested a value of 2.74 for cement pastes and mortars [48]. But for moisture transport, the interaction of pore network with water molecules is even stronger than O_2_ and CO_2_. In the previous study [37], $x_{D}$ was determined by fitting the measured apparent diffusion coefficient for three cement pastes with different w/c ratios. It was found that the value of $x_{D}$ is between 4.1 and 4.5 for cement pastes with w/c above 0.45. In this study, we choose $x_{D}=4.47$ for all experimental conditions. In Equation (10), the term ϕ(1−S) stands for the actual (unsaturated) pore space available for vapor diffusion. The terms $\phi^{x_{D}-1}$ and ${\left(1-S\right)}^{x_{D}+1}$ represent the tortuosity [5] and the liquid connectivity effects to some extent, respectively.

As for $K_{l}$, it is commonly assumed to be a function of S in the conventional models, but this does not work for the anomalous moisture transport. For capillary imbibition, a time-dependent term was introduced [29,30,31]. In this study, we consider that a time-dependent term can be used for the hygroscopic moisture transport as well; thus, $K_{l}$ is written in a product form [30,49].

(11) $$K_{l}\left(t,S\right)=K_{t}K_{s}$$

where $K_{t}=K_{t}\left(t\right)$ is a function of time alone, and $K_{S}=K_{S}\left(S\right)$ is a function of S alone.

#### 4.1.2. Time-Dependent Permeability

The basic assumptions of the time-dependent permeability are that: (1) the permeability is related to the microstructure of the porous material, and thus, if the microstructure is altered, permeability changes as well; (2) the microstructure is altered if the moisture state in the porous material changes. However, it is unclear how the microstructure changes with the moisture state. The most straightforward way is to assume that this change obeys a decay function for drying and a growth function for wetting, representing that the initial permeability decreases or increases with time and finally reaches a new stable value.

Here is a question: when does permeability start to change? If RH in the surrounding environment varies, the moisture state at different locations in the material does not change with the same pace. The change always starts from the contact surface with the ambient environment and then gradually moves towards the interior with time. For the region where moisture state does not change yet, the microstructure is stable, and thus, permeability is still the same as the initial permeability. Therefore, the concept of contact time was introduced by Hall to consider the time difference between different locations in the material after the stable moisture condition is disturbed [31]. Let us define the time when the moisture state at a given location starts to change as the initial change time $t_{c0}$. The contact time at this location is the time difference between the current time t and $t_{c0}$.

(12) $$t_{c}=t-t_{c0}$$

One may realize that the value of $t_{c0}$ is related to the time step Δt. Ideally, at a given location, after the change of the moisture condition is detected ($\left|S^{j-1}-S^{j}\right|>0$, j stands for the number of time step), the time step Δt must be reduced to satisfy $\left|S^{j-1}-S^{j}\right|\to 0$. Consequently, the time step must be very close to zero (Δt→0) if the above criterion is strictly fulfilled. This will lead to an extremely slow numerical calculation. Therefore, we intensively do not reduce the time step. Instead, the maximum time step was set as 100 s, meaning that the maximum error of the determined $t_{c0}$ is 100 s.

**Figure 4:**
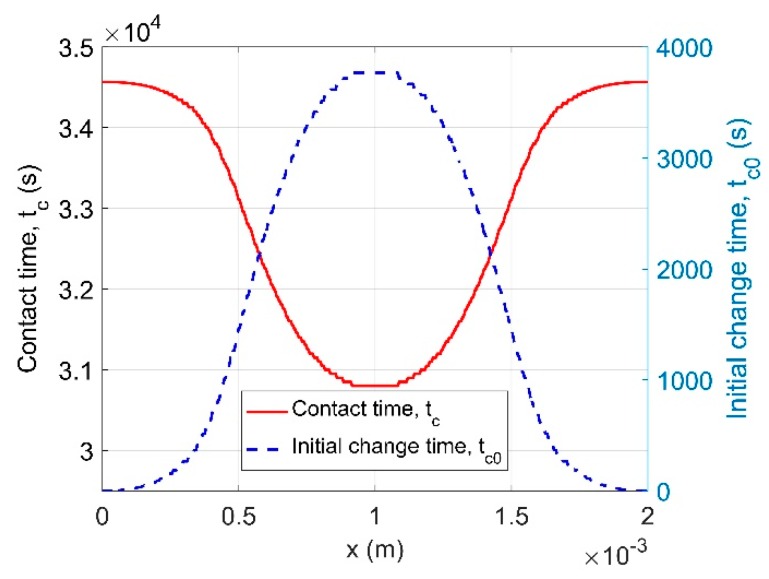
An example of contact time and initial contact time for a cylindrical specimen dried at both ends (x=0 and x=2 mm). This example is taken from the drying at 80% RH from initial 90% RH (see Section 4 for detail) and uses the exponential function to calculate $K_{t}$.

One example of contact time and initial change time is shown in Figure 4 for a cylindrical specimen that is dried at two ends. The initial change time in the regions close to the boundary is very low, indicating that moisture in these regions moves fast. Moisture must pass through these regions to reach the inner region of the specimen; therefore, we see that $t_{c0}$ quickly rises in the interior.

The contact time can be then combined with a decay or growth function to calculate the change of permeability due to the alteration of moisture state. Few functions can be used to achieve this purpose, such as a stretched exponential function suggested in [31] or a reciprocal function.

(13) $$K_{t}=K_{1}\pm K_{2}\exp\left[-{\left(\frac{t_{c}}{\tau }\right)}^{a}\right]$$

(14) $$K_{t}=K_{1}\pm \frac{K_{2}}{1+{\left(\frac{t_{c}}{\tau }\right)}^{a}}$$

In both functions, τ [s] is a time constant, and a [−] is a stretched exponent. Specification of “±” is: “+” for a drying process and “−” for a wetting process. At time zero, the initial time-dependent permeability is $K_{1}+K_{2}$. With the increase of time, the time-dependent permeability gradually decreases to $K_{1}$. Rewriting the above equations as the kinetics form, we have

(15) $$\frac{dK_{t}}{dt_{c}}=\pm K_{2}\left[-\frac{a}{\tau }{\left(\frac{t_{c}}{\tau }\right)}^{a-1}\right]\exp\left[-{\left(\frac{t_{c}}{\tau }\right)}^{a}\right]$$

(16) $$\frac{dK_{t}}{dt_{c}}=\pm K_{2}\left[-\frac{a}{\tau }{\left(\frac{t_{c}}{\tau }\right)}^{a-1}\right]{\left[1+{\left(\frac{t_{c}}{\tau }\right)}^{a}\right]}^{-2}$$

It is clear that $K_{2}$ actually is the kinetic rate constant for the change of permeability with time.

**Figure 5:**
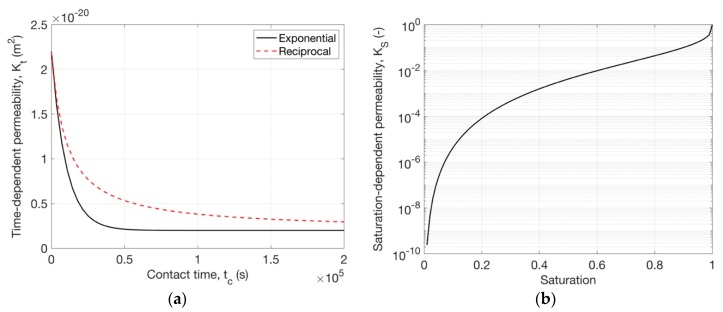
An example of kinetic permeability: (**a**) time-dependent permeability as a function of contact time for a drying process (with $K_{1}$ = 0.2 *×* 10^−20^ m^2^, $K_{1}$ = 2 *×* 10^−20^ m^2^, a = 1 and τ = 10000 s); (**b**) saturation-dependent permeability for the studied material.

As shown in Figure 5a, $K_{t}$ calculated by the exponential function changes more rapidly than that by the reciprocal function. By adjusting the parameters, the decrease of $K_{t}$ can happen immediately when the initial stable moisture state is disturbed or after a certain time, which is probably true for some cases in which the microstructure needs time to be changed by moisture.

#### 4.1.3. Saturation-Dependent Permeability

A traditional method to calculate the saturation-dependent permeability is to use the capillary pressure–saturation curve for unsaturated porous materials. Assuming a homogeneous porous material that has connected pores with cylindrical shapes, Mualem proposed the following equation for the saturation-dependent permeability $K_{S}$ [50].

(17) $$K_{S}=S^{l}{\left[\frac{\int_{0}^{S}\frac{1}{P_{c}\left(x\right)}dx}{\int_{0}^{1}\frac{1}{P_{c}\left(x\right)}dx}\right]}^{2}$$

There are three implications in this equation. First, the permeability is a reciprocal function of capillary pressure $P_{c}$ in an unsaturated porous material. With the decrease of capillary pressure, $K_{S}$ increases as more pores are filled with liquid water. Second, the square means that pores in the porous material are assumed to be cylinders, and the pore body and the pore throat of each group of pores have the same size. Third, the term $S^{l}$ is a correction factor that accounts for the influence of tortuosity, multiple interconnections, etc.

To use Equation (17), it should be combined with an equation for the capillary pressure–saturation curves. However, Equation (17) is in an integral form which is not easy to be implemented into a numerical model. It would be useful to turn it into a normal analytical function by choosing an appropriate equation for the capillary pressure curve ($S-P_{c}$). In the literature, very few equations can work in this way. The equation proposed by van Genuchten is one of them. By inserting Equation (9) into Equation (17), we have the van Genuchten–Mualem (VGM) equation [45].

(18) $$K_{S}=S^{l}{\left[1-{\left(1-S^{1/m}\right)}^{m}\right]}^{2}$$

The parameter l may vary with different porous materials. The value of 1/2 is acceptable for most soils [36,45] and also used for cementitious materials [11,51,52]. However, many studies argue that l for cement-based materials is very different to soils. A study reported that −1/2 is the best value for the three types of concretes [53]. Another study tested cement paste and mortar with four water-to-cement ratios, and they fitted l for each of them [54]. They found that the value of l is in a narrow range from −2.5 to −3. Therefore, we chose l=−3 for the studied cement pastes in this paper. Figure 5b shows that when saturation decreases from 1 the saturation-dependent permeability drops sharply.

### 4.2. Fractional Derivative Model (FDM) of Diffusion Model

As aforementioned, when Fick’s law with a constant diffusion coefficient does not work, the nonlinear modelling with time-/saturation-dependent permeability/diffusivity can be used. However, Kuntz and Lavallee [55] believed that the short-term anomaly is due to the deviation from moisture flux-gradient proportionally, meaning that the normal derivative of Fick’s law is not valid for moisture movement in porous media. Alternatively, in recent years, the fractal or fractional derivatives of diffusion equations have been found effective in modelling anomalous moisture diffusion in cement-based materials [56,57]. The normal (Fickian) diffusion of moisture follows the Brownian motion in which the moisture content is proportional to the first power of time, while in anomalous diffusion, the first power of time cannot be held anymore. The diffusion becomes a continuous-time random walk, which corresponds to the fractional diffusion equations [25]. These models are of a phenomenological description of moisture transport that may not reflect the actual physical mechanism behind them. The diffusion equation thus can be written as

(19) $$\frac{\partial^{\beta }S}{\partial t^{\beta }}=D_{f}\frac{\partial^{2}S}{\partial x^{2}}$$

where β [−] is a dimensionless exponent, so-called fractional order. With β = 1, Equation (19) turns into a normal diffusion equation, β< 1 for sub-diffusion, and β > 1 for the super-diffusion. $D_{f}$ [m^2^/s^β^] is the fractional diffusion coefficient.

For a specimen with a planar geometry (e.g., a plane sheet), uniform initial moisture content and the same external RH at both ends, the mass ratio (MR) of the specimen during moisture transport can be calculated by an equation analogy of the solution to the normal diffusion [58].

(20) $$\mathrm{MR}=\frac{m\left(t\right)-m_{e}}{m_{0}-m_{e}}=\frac{8}{\pi^{2}}\overset{\infty }{\underset{n=0}{\sum }}\left[\frac{1}{{\left(2n+1\right)}^{2}}E_{\beta }\left(-D_{f}{\left(\frac{\left(2n+1\right)\pi }{2L}\right)}^{2}t^{\beta }\right)\right]$$

where $E_{\beta }$ is the Mittag–Leffler function. If $E_{\beta }\to 1$, $E_{\beta }$ converges to an exponential function. For the long drying time, Equation (20) can be further reduced to the following equation [50,59].

(21) $$\mathrm{MR}=\frac{8}{\pi^{2}}\exp\left[-D_{f}{\left(\frac{\pi }{2L}\right)}^{2}t^{\beta }\right]$$

Let

(22) $$k={\left[D_{f}{\left(\frac{\pi }{2L}\right)}^{2}\right]}^{-1/\beta }$$

and then Equation (21) is written as

(23) $$\mathrm{MR}=\frac{8}{\pi^{2}}\exp\left[-{\left(\frac{t}{k}\right)}^{\beta }\right]$$

Equation (23) is a version of Weibull function with a scale factor of $8/\pi^{2}$. The Weibull function has been commonly used to fit the drying kinetics of irregular porous materials, such as foods [60,61] and cement-based materials [19,42]. The standard form of the Weibull function is written as

(24) $$\mathrm{MR}=\exp\left[-{\left(\frac{t}{k}\right)}^{\beta }\right]$$

The scale factor in the standard Weibull function is 1. For the purpose of fitting experimental data, some studies [27] let the scale factor be a free parameter and then Equation (23) becomes

(25) $$\mathrm{MR}=k_{1}\exp\left[-{\left(\frac{t}{k}\right)}^{\beta }\right]$$

Presumably, Equation (25) would be able to provide better fitting results, but it is different from Equation (21). Similar to Equation (15), the kinetic form of Equation (25) is

(26) $$\frac{d\;\mathrm{MR}}{dt}=k_{1}\left[-\frac{\beta }{\tau }{\left(\frac{t}{k}\right)}^{\beta -1}\right]\exp\left[-{\left(\frac{t}{k}\right)}^{\beta }\right]$$

with $k_{1}$ as the kinetic rate constant of the MR changing with time.

Therefore, this study will compare five models, which are two versions of the proposed model and three phenomenological models (two simplified solutions to the FDM in Equations (23) and (25) and the Weibull function in Equation (24)). Parameters in these models that need to be determined by fitting the measured data are shown in Table 1.

### 4.3. Experimental Data

The proposed model needs the measured sorption isotherm as input data and the measured mass change curve (or MR curve) for the model validation. Most of anomalous moisture transport data for the hygroscopic range reported in the literature cannot meet the requirement. Saeidpour and Wadsö [16] reported anomalous mass change kinetics when cement paste specimens were subject to drying or wetting in a Dynamic Vapor Sorption analyzer (DVS 1000 from Surface Measurement System Ltd., UK). Specimens were prepared from CEM I–R32.5 R according to the European standard EN 197-1, with 0.5 water-to-cement ratio without any admixtures. The materials mixing procedure followed the European standard EN 196-1. The cylindrical specimens were cast in small stainless-steel tubes (inner diameter 5.5 mm and sample length 2.0 mm) with a slight vibration. Both ends were sealed with polyethylene films and then covered with glass chips so that the flat surfaces could be obtained [16]. Then, they were kept in a tight container at room temperature for about 3 months for curing. Drying and wetting tests were carried out on different specimens, which may cause high data variations.

Samples for the sorption isotherm were prepared with the same cement and mix design but in different geometry from the drying and wetting tests. The cement paste was firstly cast in the plastic flask (diameter 7 cm and height 1 cm) and sealed for three months (see details reported in [36]). A part of the large cylinder was crushed and vacuum saturated. A few small pieces (~30 mg in total) were used to measure the sorption isotherm in the DVS.

### 4.4. Application Procedure

The moisture transport model proposed in this study was implemented by a finite differential method [62]. The model can provide different sets of data. Here, the mass change with time m(t) was compared with measured data. The nonlinear least squares method was used to calculate the squares of difference between simulated and measured data. Since the decay and growth functions (see Equations (13) and (15)) for different drying or wetting conditions vary, for each measurement, the fitting was done separately. The minimization of the sum of squares was performed by the Levenberg–Marquart algorithm. For the phenomenological models (Equations (23), (24), and (25)), the equation was directly fitted with experimental curves by using the nonlinear least squares method.

The accuracy of fitting is related to the number of parameters in a model. More parameters usually yield a higher value of determination coefficient $R^{2}$ but low robustness and low sensitivity to parameters. To take into account the effect of the number of parameters, we use an adjusted $R^{2}$ [63,64] to assess the goodness of fitting.

(27) $$R_{adj}^{2}=\frac{\left(n-1\right)R^{2}-\left(p-1\right)}{n-p}$$

where n is the number of measured data, and p is the number of parameters. For the proposed model and Equation (26), p=4 and for Weibull function (Equation (24)) and Equation (23), p=3.

## 5. Conclusions

This study considered permeability as a kinetic variable, which can be divided into two parts, one associated with saturation and one related to the contact time. The time-dependence can be simply formulated by a decay or growth function. The saturation-dependence was calculated by the VGM model. The proposed model was compared with the simplified solutions to a fractional derivative model of anomalous diffusion and the Weibull function, which is often used to estimate the drying kinetics of various porous materials. All these models were validated by data obtained from experiments performed on cement pastes exposed to the hygroscopic environment. We found that:

- The proposed model can improve the simulation results compared with the conventional model and the dual-permeability model. The main reason is that the time-dependent permeability is used in the model.
- The time-dependent permeability can be calculated either by an exponential function or by a reciprocal function. Both functions provided very similar results.
- The two simplified solutions to the fractional derivative model were not able to provide appropriate mass change curves because their curves do not pass through the origin.
- Weibull function can provide results as good as the proposed model, but the empirical equation lacks physical meanings. It may be useful for determining the final mass of a specimen when measuring the sorption isotherm.

## Figures and Tables

**Table 1:** Fitted parameters for compared models.

Models	Parameters	RH50-60	RH60-70	RH80-70	RH90-80
Proposed (Exponential $K_{t}$, Equation (13))	$K_{1}$( × 10^−22^)	163.3	144.5	1.0	0.6
	$K_{2}$( × 10^−22^)	9.4	5.7	3.5	11.4
	a	5.13 × 10^−4^	5.43 × 10^−5^	6.29 × 10^−4^	8.15 × 10^−3^
	τ	4.39	3.31	8.78	0.89
	$R_{adj}^{2}$	0.9983	0.9966	0.9963	0.9988
Proposed (Reciprocal $K_{t}$, Equation (14))	$K_{1}$( × 10^−22^)	171.3	144.8	0.8	0.6
	$K_{2}$( × 10^−22^)	13.9	6.1	3.6	9.0
	a	6.33 × 10^−4^	8.65 × 10^−5^	6.00 × 10^−4^	7.68 × 10^−3^
	τ	3.10	2.57	7.96	2.24
	$R_{adj}^{2}$	0.9984	0.9966	0.9963	0.9988
Weibull (Equation (24))	$m_{e}$	1.01	1.00	0.79	0.94
	k	8360	16134	38983	14129
	β	0.71	0.61	0.77	0.45
	$R_{adj}^{2}$	0.9988	0.9996	0.9996	0.9932
Equation (23) (FDM)	$m_{e}$	1.00	0.97	0.73	0.90
	k	11343	20532	41189	18405
	β	0.91	0.83	1.15	0.60
	$R_{adj}^{2}$	0.9965	0.9931	0.9897	0.9831
	$D_{f}$	3.21 × 10^−10^	4.18 × 10^−10^	7.68 × 10^−12^	4.32 × 10^−9^
Equation (25) (FDM)	$m_{e}$	1.01	1.01	0.80	1.02
	k	9250	15589	39042	9489
	β	0.76	0.59	0.74	0.32
	$k_{1}$	0.94	1.03	1.02	1.28
	$R_{adj}^{2}$	0.9983	0.9996	0.9997	0.9964
